# Rapid profiling of carcinogenic types of *Helicobacter pylori* infection via deep learning analysis of label-free SERS spectra of human serum

**DOI:** 10.1016/j.csbj.2024.09.008

**Published:** 2024-09-16

**Authors:** Fen Li, Yu-Ting Si, Jia-Wei Tang, Zeeshan Umar, Xue-Song Xiong, Jin-Ting Wang, Quan Yuan, Alfred Chin Yen Tay, Eng Guan Chua, Li Zhang, Barry J. Marshall, Wei-Xuan Yang, Bing Gu, Liang Wang

**Affiliations:** a Department of Laboratory Medicine, Huai'an Hospital Affiliated to Yangzhou University (The Fifth People's Hospital of Huai'an), Huai'an, Jiangsu, China; b Laboratory Medicine, Guangdong Provincial People’s Hospital (Guangdong Academy of Medical Sciences), Southern Medical University, Guangzhou, Guangdong, China; c Medical Technology School, Xuzhou Medical University, Xuzhou, Jiangsu, China; d Marshall Laboratory of Biomedical Engineering, International Cancer Center, School of Biomedical Engineering, Shenzhen University Medical School, Shenzhen, Guangdong, China; e Department of Gastroenterology, Huai'an Hospital Affiliated to Yangzhou University (The Fifth People's Hospital of Huai'an), Huai'an, Jiangsu, China; f Department of Intelligent Medical Engineering, School of Medical Informatics and Engineering, Xuzhou Medical University, Xuzhou, Jiangsu, China; g The Marshall Centre for Infectious Diseases Research and Training, University of Western Australia, Perth, Western Australia, Australia; h Marshall Medical Research Center, Fifth Affiliated Hospital of Zhengzhou University, Zhengzhou, Henan, China; i Marshall International Digestive Diseases Hospital, Zhengzhou University, Zhengzhou, Henan, China; j School of Biotechnology and Biomolecular Sciences, University of New South Wales, Sydney, New South Wales 2052, Australia; k Division of Microbiology and Immunology, School of Biomedical Sciences, The University of Western Australia, Crawley, Western Australia, Australia; l Centre for Precision Health, School of Medical and Health Sciences, Edith Cowan University, Perth, Western Australia, Australia; m School of Agriculture and Food Sustainability, University of Queensland, Brisbane, Queensland, Australia

**Keywords:** *Helicobacter pylori*, Surface-enhanced Raman spectrometry, Deep learning algorithm, Gastric cancer, Serum antibody, Carcinogenic toxin

## Abstract

WHO classified *Helicobacter pylori* as a Group I carcinogen for gastric cancer as early as 1994. However, despite the high prevalence of *H. pylori* infection, only about 3 % of infected individuals eventually develop gastric cancer, with the highly virulent *H. pylori* strains expressing cytotoxin-associated protein (CagA) and vacuolating cytotoxin (VacA) being critical factors in gastric carcinogenesis. It is well known that *H. pylori* infection is divided into two types in terms of the presence and absence of CagA and VacA toxins in serum, that is, carcinogenic Type I infection (CagA+/VacA+, CagA+/VacA-, CagA-/VacA+) and non-carcinogenic Type II infection (CagA-/VacA-). Currently, detecting the two carcinogenic toxins in active modes is mainly done by diagnosing their serological antibodies. However, the method is restricted by expensive reagents and intricate procedures. Therefore, establishing a rapid, accurate, and cost-effective way for serological profiling of carcinogenic *H. pylori* infection holds significant implications for effectively guiding *H. pylori* eradication and gastric cancer prevention. In this study, we developed a novel method by combining surface-enhanced Raman spectroscopy with the deep learning algorithm convolutional neural network to create a model for distinguishing between serum samples with Type I and Type II *H. pylori* infections. This method holds the potential to facilitate rapid screening of *H. pylori* infections with high risks of carcinogenesis at the population level, which can have long-term benefits in reducing gastric cancer incidence when used for guiding the eradication of *H. pylori* infections.

## Graphical Abstract

**Figure:**
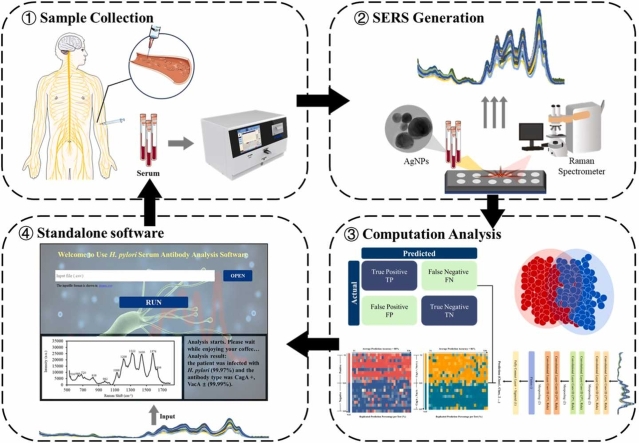

## 1 Introduction

*Helicobacter pylori* is one of the most common human infectious agents globally. A recent meta-analysis examining the prevalence of *H. pylori* infections across 62 countries found that 48.5 % of the individuals tested positive for the infection [1]. *H. pylori* exhibits greater prevalence in Asia, South America, and Africa compared to North America and Oceania [1], [2]. For instance, the infection rate in Australia is 24.6 %, while Nigeria's is 87.7 % [3]. Infection rates are notably elevated in low- and middle-income nations compared to high-income countries [4]. These findings imply a robust association with socio-economic factors that can either enhance or diminish the transmission of *H. pylori* infections. Enhanced socio-economic conditions, marked by improved household sanitation, proper sewage disposal, and clean water sources, significantly limit the spread of *H. pylori* [5]. China is a country with a high prevalence of *H. pylori* infection. Ren et al. meta-analyzed 412 eligible studies and assessed the prevalence of *H. pylori* infection in mainland China as 44.2 % [6]. Based on these findings, it is evident that *H. pylori* infection remains a significant global health problem. Within this context, an international consensus has been reached to recommend including universal screening and eradication of *H. pylori* infection in national health priorities to optimize healthcare resources [7].

Long-term infection with *H. pylori* is associated with many gastric diseases, such as chronic gastritis, peptic ulcer disease, gastric intestinal metaplasia, and gastric cancer or mucosa-associated lymphoid tissue (MALT) lymphoma[8], [9]. Therefore, early diagnosis and effective treatment of infection caused by this pathogen are essential. Diagnosing *H. pylori* can be divided into two main categories: invasive and non-invasive, each presenting advantages and disadvantages [10]. Direct (invasive) histological testing of gastric mucosal biopsy specimens is preferred for patients requiring endoscopy. With appropriate sampling and interpretation, histological testing for *H. pylori* in gastric tissues has a sensitivity of approximately 90 % and a specificity of 95–100 % [11].

In contrast, non-invasive testing methods are recommended for those without endoscopy but with symptoms generally associated with *H. pylori* infection. The urea breath test (UBT) is the most popular non-invasive diagnostic test. According to a 2015 meta-analysis on patients with dyspepsia, the UBT demonstrates a diagnostic sensitivity of 96 % and a specificity of 93 % [12]. As an alternative non-invasive method, stool antigen testing (SAT) also shows an excellent sensitivity and specificity of 94 % and 97 %, respectively [13]. The detection of *H. pylori* antigens in fecal samples is primarily accomplished by SAT through the utilization of enzyme immunoassay (EIA) or immunochromatographic assay (ICA). Furthermore, serological tests for detecting antibodies against *H. pylori* antigens provide quick and easy diagnostic options and are often used in epidemiological studies. However, due to differences in strains and antigens in different geographic regions, serological tests need to be validated in the appropriate area [13], and reliable thresholds for serological tests must be established on a case-by-case basis [14]. In addition, as serological tests cannot differentiate between active and previous infections, additional confirmatory tests are necessary after undergoing eradication therapy.

While approximately 80 % of *H. pylori* infections are asymptomatic, all infected individuals develop different types of gastritis [15], [16], which results from a complex interaction of bacterial virulence factors, host genetics, and environmental factors. Among these, vacuolating cytotoxin A (VacA) and cytotoxin-associated gene A (CagA) are two important virulence factors of *H. pylori* [17]. VacA is an oligomeric self-transporter protein toxin that forms anion-selective membrane channels [18]. It enables *H. pylori* to escape the host immune system mainly by inducing epithelial cell vacuolization [19] and autophagy and inhibiting lymphocyte proliferation [20], leading to chronic infection. CagA is encoded by the Cag Pathogenicity Island (PAI) and is injected into host epithelial cells [21]. Both phosphorylated and unphosphorylated forms of CagA proteins regulate signaling pathways by interacting with multiple host proteins [22], [23], [24], which may ultimately lead to carcinogenesis. Abdullah et al. revealed synergistic effects between VacA and CagA, suggesting that CagA may accumulate in VacA-induced damaged autophagosomes [25]. The study conducted by Karami et al. has provided additional evidence that the risk of gastric cancer was nearly five times higher in patients infected by *H. pylori* strains expressing both CagA and VacA proteins, as confirmed by serological tests (ELISA and Western blot). These findings highlight the importance of CagA and VacA proteins as reliable markers of the risk of developing gastric cancer, offering the potential to be used to identify individuals with high-risk *H. pylori* infections by non-invasive serological testing. However, in settings with limited resources where conducting serological tests is difficult due to the lack of specialized personnel, advanced equipment, and cost-effectiveness, there is a growing urgency for a rapid, simple, and cheap diagnostic method to determine antibody typing against its major virulence factors during *H. pylori* infection.

Surface-enhanced Raman Scattering (SERS) has garnered substantial recent attention in sensing applications due to its ability to amplify the Raman effect signals for molecules adsorbed on nanostructures. The convergence of this amplification effectively positions SERS as an up-and-coming option for various applications. In recent years, SERS has been widely applied in clinical settings, successfully identifying biomolecules [26], pathogenic microorganisms [27], body fluid samples [28], and cancer tissues [29]. However, the differences between SERS fingerprints are difficult to observe with the naked eye when detecting complex components, which poses a challenge to extracting and interpreting detailed sample information. The emergence of machine learning methods has introduced a new avenue for addressing these challenges [30]. In a study on lung cancer screening, Fan et al. proposed a machine learning-driven blood-SERS analytical technique utilizing a self-position detection platform to rapidly and accurately differentiate lung cancer from benign lesions [31]. The AdaBoost classifier achieved a diagnostic accuracy of 96.3 % for distinguishing lung cancer from benign and normal cases [31]. Lin et al. studied early cancer screening through serum SERS analysis. A deep learning model was employed to analyze the SERS spectra of 203 healthy volunteers, 77 leukemia M5 patients, 94 hepatitis B virus patients, and 321 breast cancer patients, achieving a classification accuracy of 100 % [32]. Therefore, combining SERS and machine learning algorithms is a powerful strategy for assisting in disease diagnosis and can be utilized to develop high-throughput, rapid, and label-free disease screening tools.

In this study, we report the exploration of human serum using SERS technology. We successfully distinguished Hp-negative and Hp-positive serum samples, as well as Type I (CagA +, VacA ±) and Type II (CagA -, VacA-) *H. pylori* infections using SERS combined with a deep learning algorithm convolutional neural network (CNN), in a rapid, non-disruptive, and label-free manner. Subsequently, the generated model was employed to detect unknown samples, exploring the feasibility of implementing SERS strategies in clinical settings. Finally, we developed a serum SERS spectral analysis software for detecting *H. pylori* infection status and the two immune-response types, which can automatically analyze input spectra and provide prediction results along with accuracy values. The proposed method and further research could give convenient measures for diagnosing and screening *H. pylori* in resource-limited settings.

## 2 Material and methods

### 2.1 Serum sample collection and detection

4 mL of fasting venous whole blood samples were collected from the study subjects in the morning, which was then centrifuged at 4000 r/min for 5 min using a bench-top centrifuge (model type: XZ-P5, Changsha Xiangzhi Centrifuge Instrument Co., Ltd., China). The serum was separated from the blood samples and stored at 4 ℃ for subsequent measurements. Quantum dot immunofluorescence assay was employed for qualitative detection of *H. pylori* antibodies of Urease, Cytotoxin-associated protein A (CagA), and vacuotoxin-associated protein A (VacA) in human serum samples (Chongqing Xin Saiya Biotechnology Co., Ltd., China). During the testing process, 80 μL of serum sample was vertically added to the sample well, the detection card was inserted into the dry fluorescence immunoanalyzer AFS2000A (AmonMedCAS, China), and automatic timing was selected. The testing was completed within 15 min, and the results were recorded. Guidelines for determining *H. pylori* infection status and serologic typing are listed below 1) Urease < 8 (non-*H. pylori* infection); 2) Urease ≥ 8 and either CagA ≥ 6 or VacA ≥ 4 (Type I *H. pylori* infection); 3) Urease ≥ 8 and CagA < 6 and VacA < 4 (Type II *H. pylori* infection). The baseline information of participants was provided in Supplementary Table S1 and Supplementary Table S2. The sample collections and experimental procedures were conducted according to the approved protocols of the Huai'an Fifth People's Hospital Ethics Committee. Informed consent was obtained from all participants involved in this clinical study, and the acquisition of serum samples was carried out following the approval of the Ethics Committee at Huai'an Fifth People's Hospital (HAWY-KY-2023–006-01). All experiments strictly adhered to the guidelines and regulations set forth by the Ethics Committee at Huai'an Fifth People's Hospital.

### 2.2 Silver nanoparticle preparation

The preparation procedure of silver nanoparticles follows previously published studies [28]. In particular, a clean and sterile Erlenmeyer flask, pre-filled with 200 mL of deionized water (ddH_2_O), was supplemented with 33.72 mg of AgNO_3_ (SinoPharm Chemical Reagent Co., Ltd., China). The flask was heated on a magnetic stirrer MS-H-ProT (DLAB Pty. Ltd., China) until boiling. Subsequently, 8 mL of a 1 % sodium citrate solution (SinoPharm Chemical Reagent Co., Ltd., China) was added to the boiling solution while stirring at 650 r/min. After heating for 40 min, stirring was stopped, and the solution was left to cool at room temperature. The solution was then refilled with ddH_2_O to a total volume of 200 mL. To obtain a uniform milky gray solution with negatively charged AgNPs, 1 mL of the solution was transferred to a 1.5 mL Eppendorf (EP) tube and centrifuged at 7000 r/min for 7 min using a Sorvall TM Legend TM Micro 21 Centrifuge. The supernatant was discarded, and the pellet was resuspended in 100 μL of ddH_2_O. The resulting solution was in uniform milky gray color with negatively charged AgNPs stored in the dark at 4 °C to ensure long-term use.

### 2.3 SERS spectral generation

SERS spectra of the silver nanoparticle-serum mixture were collected using an Anton Paar Cora100™ Raman spectrometer (Anton Paar Shanghai Trading Co., Ltd., China) with a 785 nm laser. In the experiments, the laser was operated at 25 mW, the spectral resolution was set to 1 nm, the spectral wave number resolution was 10 cm^−1^, and the instrument covered a Raman shift range of 400–2300 cm^−1^. For the calibration of all SERS spectra, the Raman peak at 520 cm^−1^ was used as the reference peak (silicon wafer), and the integration time was employed to remove dark current. To prepare the samples, 20 μL AgNPs and 20 μL serum were mixed for 5 s using a vortex mixer. Then, 30 μL of the mixture was dropped onto a silicon wafer and allowed to dry naturally. This study collected spectra from serum samples of 50 *H. pylori*-positive (Hp-positive) and *H. pylori*-negative (Hp-negative) participants and 50 Type I and Type II participants. Each participant had 100 SERS spectra collected individually.

### 2.4 Average SERS spectra and deconvolution analysis

To examine the overall distribution trend of serum Raman spectra, we generated the average Raman spectra for each category of serum SERS spectra. We calculated the moderate intensity of all spectra at each Raman shift for specific samples. The spectra standard deviation (SD) was also calculated to assess the data dispersion. Spectral visualization was accomplished in Origin software (version 2019b, OriginLab, United States), and the *fit peaks pro* function was utilized for automated fitting characteristic peaks of serum spectra. Given the high resemblance observed among the various types of serum SERS spectra, we proceeded with spectral deconvolution of the average Raman spectra to explore minute differences between the ranges. The *Vogit* function in Origin, which represents the convolution of *Lorentzian* and *Gaussian* density, was used to extract detailed information from each spectral characteristic peak. The *Gaussian* width and *Lorentzian* width values were set to 1 for all distinct peaks, and then the fitting process continued until convergence. Detailed methods are provided in Supplementary Methods.

### 2.5 Clustering analysis of SERS spectra from serum samples

Orthogonal Partial Least Squares Discriminant Analysis (OPLS-DA) was employed to extract relevant information from the spectral data to explore the inherent differences among serum SERS spectra. Specifically, all SERS data was imported into SIMCA software (Umetrics, Sweden). OPLS-DA was selected as the model type, and then Autofit to fit the model. The model’s fit degree to the input matrix was measured using the explained variance (R2X), while the fit to the output matrix was assessed using the predicted variance (R2Y). Additionally, Q2 was employed as a metric to evaluate the cross-validated predictability, representing the model’s ability to predict unknown samples. All three metrics range from 0 to 1, with values closer to 1 indicating better model performance. It is important to note that during the clustering process, we observe the dispersion of spectral sample points in the OPLS-DA coordinate system to identify and exclude extreme outliers. This step enables us to remove data points significantly deviate from the general pattern, ensuring more reliable and robust clustering results.

### 2.6 Supervised machine learning analysis of SERS spectra

After removing outliers, all spectral data were normalized to eliminate overall intensity differences (Supplementary Methods). This study used six supervised machine learning algorithms to discriminate *H. pylori* infection status and serum antibody typing. These algorithms include five classical machine learning (ML) algorithms: Adaptive Boosting (AdaBoost), Decision Tree (DT), Linear Discrimination Analysis (LDA), Random Forest (RF), Support Vector Machine (SVM), and one deep learning (DL) algorithms: Convolutional Neural Network. We divided the patients, randomly selecting 70 % of the patients as the training and validation set and 30 % as the test set to avoid data leakage. These ML algorithms were implemented by invoking the corresponding functions from the SciKit-Kearn version 0.21.3 library. Before training ML models, the parameter ranges were set in advance, and the *GridSearchCV* function was used to train and tune the model parameters to achieve the best-fitting effect of each model. For details of these parameters, please refer to Supplementary Table S3 and Supplementary Table S4.

Due to the high feature dimensionality and slight variations of the serum SERS spectral, traditional ML algorithms may need help to predict the spectra accurately [33]. To address this issue, we employed a CNN known for its excellent feature extraction capabilities to analyze the serum SERS spectra. The framework of the CNN model mainly consists of an Input Layer, Convolutional Layer, Maxpooling Layer, and Fully connected Layer. The *input_shape* of the Input layer was (636, 1); convolutional Layers were used to learn the intrinsic feature representation of the SERS spectral, and nonlinear features were detected through the application of the Rectified Linear Unit (ReLU) activation function after each convolutional layer. The convolution kernel was set to 3 * 1 and 5 * 1, while the size of filters was set to 8, 16, 32, and 64, respectively. MaxPooling layers were used to compress the extracted feature information, enabling the network to capture a broader range of features. This study connected a MaxPooling layer after each convolutional layer, and the pool size was set to 3. Following multiple convolutional and pooling layers, the global information was fed into the Fully Connected Layer for classification. The activation function *Softmax* was applied in the Fully connected Layer for classification purposes. The CNN framework was developed based on Python (version 3.7.4), PyCharm (version 2019.3.3, Community Edition), TensorFlow (version 2.4.1), and Keras (version 2.4.3).

### 2.7 Evaluation of supervised learning algorithms

Several metrics were computed for each model to evaluate the recognition and prediction capabilities of different supervised learning algorithms. These metrics include accuracy, precision, recall, F1 score, 5-fold cross-validation, and area under the curve (AUC). In particular, accuracy (*accuracy_score*) represents the proportion of correctly predicted samples to the total number of samples. At the same time, precision (*precision_score*) indicates the proportion of actual positive samples among all samples predicted as positive by the model. In contrast to precision, recall (sensitivity, *recall_score*) is used to determine the proportion of samples predicted as positive by the model out of all actual positive samples. The F1-score (*f1_score*) is the harmonic mean of precision and recall used to assess the model's performance comprehensively. Specificity determines the proportion of samples predicted as negative by the model out of all actual negative samples. A 5-fold cross-validation (CV) method was employed to prevent model overfitting during training. The training dataset was divided into five equally sized subsets using the *cross_val_score* function with the *cv* parameter set to 5. Receiver operating characteristic (ROC) curves were generated using the roc_curve process, and the area under the curve (AUC) value was computed using the roc_auc_score function. These evaluations provided insights into model performance and discriminative capabilities across different thresholds. Additionally, we plotted a confusion matrix for the best-performing models in differentiating *H. pylori* infection status and serum antibody typing tasks. The confusion matrices were generated using the *confusion_matrix* function, providing a detailed representation of the model's predictions for different serum SERS spectra in matrix form.

### 2.8 Data interpretability

Addressing the interpretability of deep learning algorithms and providing clear evidence to explain decision outcomes are effective methods for improving model reliability. In this study, we aim to demonstrate the decision process of the CNN model on SERS fingerprints. We introduce the Grad-CAM algorithm to observe the weight distribution of the CNN. Specifically, *tf.keras.models.Model* was employed to invoke the generated CNN model, the *tf.GradientTape* method was conducted to calculate the gradient vector of the last convolutional layer (*conv1d_5*, layer=5), and the channel mean was calculated using the *tf.reduce_mean* method. The Grad-CAM of the target SERS was generated according to the gradient vector and the channel mean.

### 2.9 Double-blind validation of model performance

To assess the model's predictive ability on unknown samples, we conducted two validation tasks regarding the infection status of *H. pylori* and the antibody typing. An additional 20 Hp-positive and Hp-negative participants and 19 Type I and Type II participants were recruited as external samples. Serum samples of all participants were collected using the same method as the tested participants, with 30 SERS signals collected from each participant for external validation of the model. The baseline information of the participants is provided in Supplementary Tables S7 and S8. All participants were informed about the purpose and procedure of this study, and they signed consent forms to participate. The obtained spectral matrices from the participants were input into the optimal diagnostic model, and the judgment results of the model for each spectrum were displayed in the form of a heatmap. All participants' true infection status and antibody typing were qualitatively assessed by quantum dot immunofluorescence to evaluate the presence of *H. pylori* urease, CagA, and VacA antibodies in the serum samples.

### 2.10 Software development

The standalone analytical software was developed using PyCharm and PyQt5 to create a convenient tool for SERS spectral analysis for users without a computational background. Within just a few clicks, users can conveniently achieve its functionalities. It is accessible free of charge on the GitHub webpage: https://github.com/4forfull/1DCNN_Serum. All software functions were implemented by calling self-written packages. Specifically, the *find_peaks_cwt* process embedded in the "*OPEN*" button was used to fit spectral characteristic peaks, which were then labeled on the average spectrum using the *ax.text* method. A pre-trained model file (.h5) was embedded in the “RUN” button to identify unknown SERS spectra, automatically predicting infection status and typing for unknown serum samples. The prediction results were displayed in the *QTextEdit* plugin in the interface’s bottom-right corner.

## 3 Result

### 3.1 SERS spectral analysis of *H. pylori* infection

**Fig. 1:**
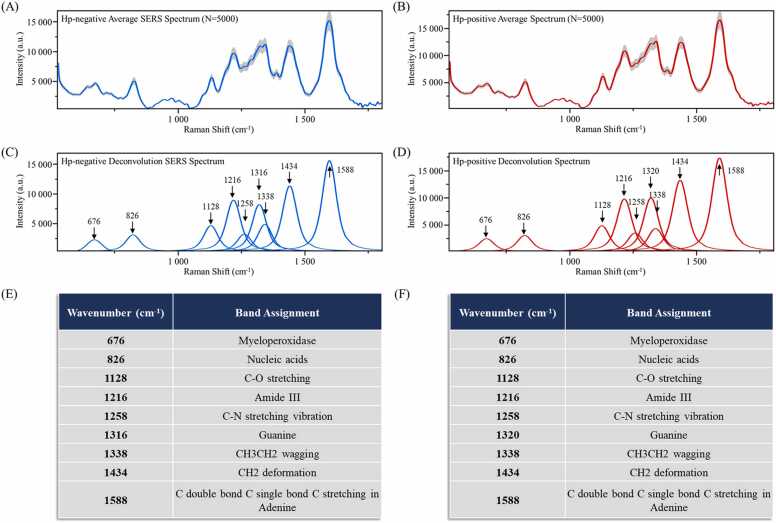
Average and deconvoluted SERS spectra of Hp-positive and Hp-negative serum samples. (A) Average SERS spectra of Hp-negative serum samples, (B) Average SERS spectra of Hp-negative serum samples. (C) Deconvoluted SERS spectra of Hp- negative serum samples, (D) Deconvoluted SERS spectra of Hp-positive serum samples. (E) Characteristic peaks of Hp-negative samples and biological significance. (F) Characteristics of Hp-positive samples and biological significance. The X-axis represents Raman shifts in the 530–1800 cm^−1^ range, while the Y-axis represents the relative Raman intensity. a.u. is an arbitrary unit, referring to the relative value of each data under the same measurement conditions.

Average SERS spectra were initially examined to assess the distribution and quality. Preliminary results indicated little difference between the average spectra of Hp-negative (Fig. 1**A**) and Hp-positive (Fig. 1**B**) serum samples. The gray shaded areas in the figure depicted the value of SD for two types of samples. The narrower the shaded error band, the smaller the differences between the same type of SERS signals. The quality assessment of SERS signals and spectral characteristic peaks are provided in Supplementary Fig. S1. Further refinement through spectral deconvolution (Fig. 1**C-D**) revealed that both types of SERS samples shared eight characteristic peaks. However, there were noticeable variations in the intensities of these peaks. In particular, the ridge at 1434 cm^−1^, attributing to CH2 deformation in proteins and lipids, exhibited the most significant discrepancy, with a difference of over 2 × 10^5^ in the peak area [34]. Bacterial infections are associated with elevated levels of serum fibrinogen and lipids [35]. Persistent infection with *H. pylori* can produce pro-inflammatory cytokines, such as C-reactive protein, interleukin (IL)−6, and IL-8, inducing chronic inflammation and immune responses. It is known that *H. pylori* infection leads to increased levels of total cholesterol, triglycerides, and high-density and low-density lipoproteins [36]. A different Raman shift was observed in the peaks associated with Guanine in Hp-negative (Fig. 1**E**) and Hp-positive (Fig. 1**F**) serum samples, corresponding to 1316 cm^−1^ [37] and 1320 cm^−1^ [38], respectively. This change may stem from alterations in the relative content and conformation of RNA/DNA during disease progression [39]. Environmental and chemical factors can induce the formation of 8-hydroxydeoxyguanosine (8OHdG) from guanine in DNA, a marker that typically shows elevated levels in *H. pylori*-associated gastric diseases [40]. Moreover, 8OHdG levels are higher in CagA-positive *H. pylori* patients compared to CagA-negative and *H. pylori*-negative patients. The results of this study indicate that the peak area under the curve differed by more than 1.5 × 10^5^. Further details on characteristic peaks were provided in Supplementary Table S5.

### 3.2 SERS spectral analysis of two serological types of *H. pylori* infection

**Fig. 2:**
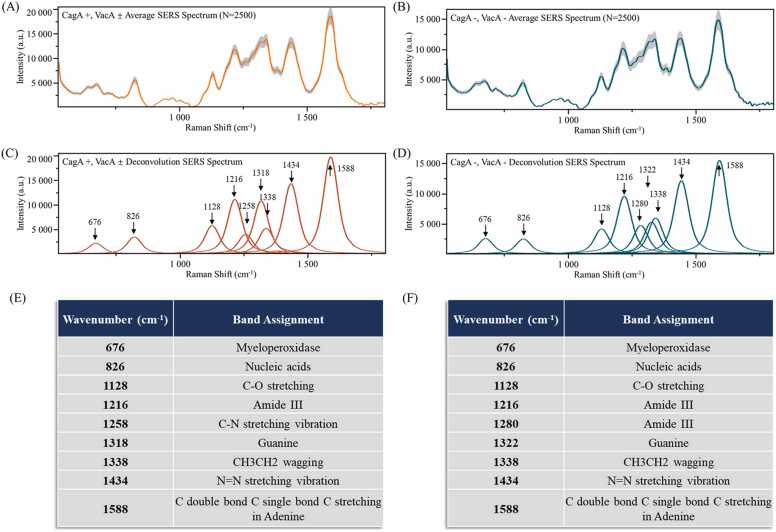
Average and deconvoluted SERS spectra of the type of serum *H. pylori*. (A) Average SERS spectra of Type I serum samples, (B) Average SERS spectra of Type II serum samples. (C) Deconvoluted SERS spectra of Type I serum samples, (D) Deconvoluted SERS spectra of Type II serum samples. (E) Characteristic peaks of Type I serum samples and biological significance. (F) Characteristics of Type II serum samples and biological significance. The X-axis represents Raman shifts in the 530–1800 cm^−1^ range, while the Y-axis represents the relative Raman intensity. a.u. is an arbitrary unit, referring to the relative value of each data under the same measurement conditions.

The SERS analysis of Type I and Type II Hp-infection revealed minimal differences in the average spectra of these two sets of human serum samples (Fig. 2**A-B**). Similarly, the SD values were displayed in the shaded area in the graph. Spectral deconvolution processing revealed the presence of seven familiar characteristic peaks in the SERS spectra of the two types of serum samples (Fig. 2**C-D**). Among these peaks, the maximal difference in relative intensity was found for the peak formed by CH_3_CH_2_ wagging at 1338 cm^−1^ [41], exceeding 7 × 10^4^. In addition to the shared characteristic peaks, the SERS spectra of Type I and Type II Hp-infection displayed two distinct differential peaks. Similar to the Hp-negative and Hp-positive serum samples, a Raman shift was observed in the peak associated with Guanine. In Type I Hp-infection, it occurred at 1318 cm^−1^ (Fig. 2**E**) [42], while in Type II Hp-infection, it occurred at 1322 cm^−1^ (Fig. 2**F**) [43]. Additionally, Type I Hp-infection exhibited a unique peak at 1258 cm^−1^, corresponding to the C-N stretching vibration [44], and Type II Hp-infection showed a peak at 1280 cm^−1^ associated with Amide III [45]. These two characteristic peaks may serve as potential criteria for distinguishing the two types of immune responses of Hp infections in serum. Further details on distinct peaks were provided in Supplementary Table S6.

### 3.3 Clustering analysis of serum SERS spectra

**Fig. 3:**
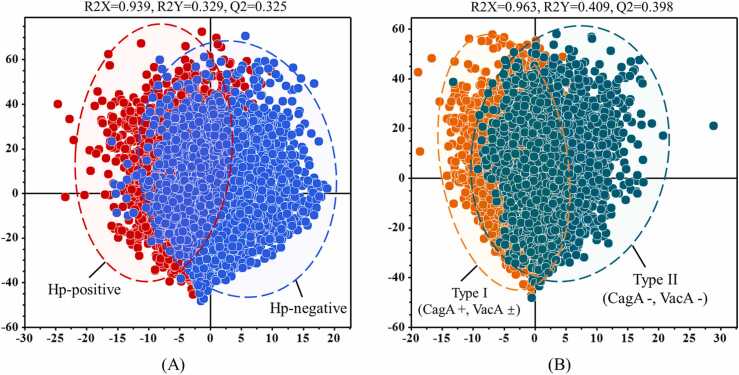
Clustering analysis of SERS spectra of serum samples via OPLS-DA. (A) Clustering scatter plot of positive and negative serum samples of Hp-infection. (B) Clustering scatter plot of serum samples with Type I and Type II Hp-infection.

After several iterations of clustering and removing extreme outliers, the performance of OPLS-DA remains stable. When identifying the infection status of serum samples in the case of *H. pylori* (Fig. 3**A**), there is an overlap between the SERS serum sample points of Hp-positive and Hp-negative examples, with R2X= 0.985, R2Y= 0.329, and Q2 = 0.325. Similarly, this overlap exists in identifying Type I and Type II antibody typing tasks (Fig. 3**B**), with R2X= 0.963, R2Y= 0.409, and Q2 = 0.398. These results indicate that OPLS-DA could not effectively distinguish between different serum samples. Therefore, it is necessary to explore advanced chemometric analysis techniques to improve the accuracy of serum SERS spectra identification.

### 3.4 Prediction capacity of supervised learning models

The comparative performance of different supervised learning algorithms in identifying serum infection is presented in Table 1. The results showed that CNN acquired a prediction accuracy of 90.37 %. This indicates that the CNN model can effectively identify the status of *H. pylori* infection in serum samples. In addition, the random forest algorithm also showed remarkable feature extraction capabilities by integrating numerous weak classifiers (*n_estimators*) and leaf node depth (*max _depth*), achieving a good recognition accuracy of 89.25 %. However, due to the non-linear relationship of serum Raman spectra, the effectiveness of the SVM (Accuracy=68.26 %)could have been better.

**Table 1:** Comparison of the predictive abilities of six supervised machine learning algorithms in the analysis of positive and negative serum SERS data.

Positive & Negative	Accuracy	Precision	Sensitivity	Specificity	F1	5Fold
**CNN**	90.37 %	90.37 %	90.35 %	91.40 %	90.37 %	89.51 %
**RF**	89.25 %	89.25 %	89.24 %	89.41 %	89.25 %	87.73 %
**DT**	78.14 %	78.14 %	78.12 %	79.44 %	78.14 %	76.71 %
**AdaBoost**	77.65 %	77.65 %	77.65 %	77.99 %	77.65 %	78.18 %
**LDA**	76.57 %	76.57 %	76.51 %	79.71 %	76.54 %	75.03 %
**SVM**	68.26 %	68.26 %	68.20 %	60.52 %	68.21 %	68.40 %

Apart from assessing the presence of *H. pylori* in the serum, we also identified the antibody types to provide more precise diagnoses and treatment plans when analyzing the serum of the infected patients. The results showed that (Table 2), the CNN model consistently achieves the highest identification results, with an accuracy of 93.18 %, underscoring its effectiveness in identifying serum SERS spectra. Furthermore, except for SVM, the prediction accuracy of all other algorithms exceeded 80 %, which may be attributed to the reduction in the number of SERS data samples.

**Table 2:** Comparison of the predictive abilities of six supervised machine learning algorithms in analyzing serum SERS data of Type I and Type II *H. pylori* infections.

Type I & Type II	Accuracy	Precision	Sensitivity	Specificity	F1	5Fold
**CNN**	93.18 %	93.18 %	93.08 %	94.97 %	93.15 %	91.25 %
**RF**	92.31 %	92.31 %	92.23 %	94.08 %	92.30 %	89.86 %
**AdaBoost**	84.54 %	84.54 %	84.47 %	86.09 %	84.53 %	82.94 %
**LDA**	83.69 %	83.69 %	83.50 %	88.31 %	83.64 %	81.27 %
**DT**	81.62 %	81.62 %	81.53 %	83.58 %	81.60 %	79.99 %
**SVM**	76.15 %	76.15 %	75.76 %	85.50 %	75.88 %	72.77 %

**Fig. 4:**
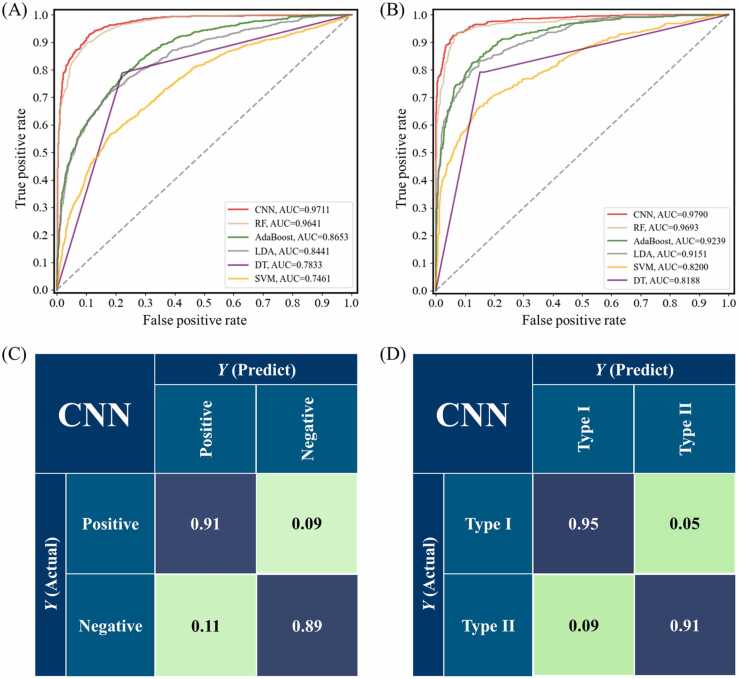
ROC curve for different supervised learning algorithms and confusion matrix of best performance model. (A-B) ROC curve, according to the comparison, CNN achieved the best performance in positive/negative and Type I/Type II classification tasks of *H. pylori* infections. (C-D) The confusion matrix number in the confusion matrix represented the percentage of correctly classified (diagonal) or misclassified (off-diagonal) spectra, respectively.

To further validate the performance of various supervised learning models in identifying serum infection status and antibody typing, ROC curves were utilized to assess the specificity and sensitivity of each model (Fig. 4**A-B**). Additionally, we evaluated the models based on the Area Under the Curve (AUC) value, which quantifies their overall performance. Meanwhile, we used confusion matrices to compare the predicted results of the optimal classification model with the true labels. In the ROC curve, the closer to the upper left area, the higher the true positive rate (TPR) and the lower the model's false positive rate (FPR). Simultaneously, we also calculated the AUC value of the area under the ROC curve to measure the models' performances. The results revealed that the CNN model exhibited the highest performance in infection identification (AUC=0.9711) and antibody typing (AUC=0.9790). Given these outcomes, we calculated and plotted the confusion matrix for the CNN model. In detecting *H. pylori* infection (Fig. 4**C**), the CNN model incorrectly classified 9 % of positive and 11 % of negative samples as positive. While performing antibody typing (Fig. 4**D**), the model misclassified 5 % of Type I and 9 % of Type II samples. These findings comprehensively assessed the supervised learning models and highlighted the CNN model's superior performance in identifying *H. pylori* infection status and profiling of carcinogenic and non-carcinogenic types of *H. pylori* infection.

### 3.5 Interpretability of deep learning analysis

**Fig. 5:**
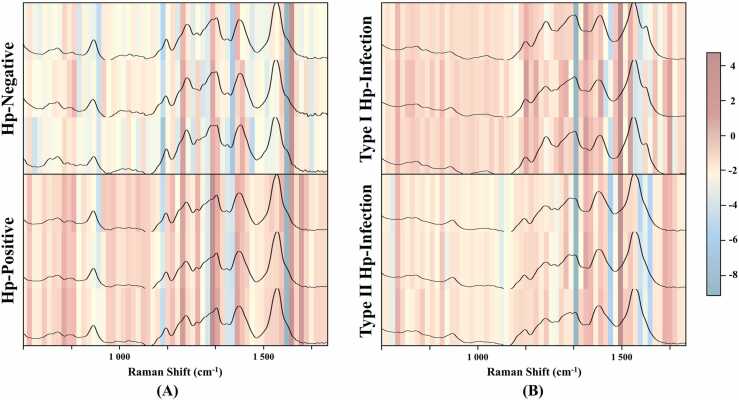
Weight distribution of CNN model. (A) Weight distribution heatmap of Hp-positive and Hp-negative serum samples. (B) Weight distribution heatmap of Type I and Type II Hp-positive serum samples. For heatmaps, the deeper the color, the more attention is allocated to the CNN model.

To improve the interpretability of the model, we employed the Grad-CAM algorithm to examine the distribution of model classification weights in each category of SERS spectra. When analyzing Hp-positive and Hp-negative serum (Fig. 5**A**), partial weightings will be allocated to essential fingerprint regions. For example, regardless of positive or negative samples, more attention was given to the characteristic peaks at 1216, 1338, and 1434 cm^−1^ and their adjacent regions. However, positive samples received more weight. Notably, not all significant characteristic peaks received attention. For instance, the peak at 1588 cm^−1^ did not play a crucial role in model decision-making when distinguishing Hp-positive from Hp-negative. Furthermore, weights were also allocated to non-characteristic fingerprint regions at 706–738 cm^−1^ and 1636–1674 cm^−1^. Similarly, in the tasks of identifying Type I and Type II serum samples of *H. pylori* infection (Fig. 5**B**), significant Raman characteristic peaks in the 1110–1480 cm^−1^ region received more attention, while four non-characteristic fingerprint regions at 600, 708, 808, and 1544 cm^−1^ also received higher weights. Additionally, unlike the negative/positive identification tasks, the model did not distribute much weight in the region beyond 1588 cm^−1^. These results indicate that the model considers a combination of multiple characteristic peaks in the decision-making process rather than solely relying on significant ones.

### 3.6 Validation analysis of the CNN model

**Fig. 6:**
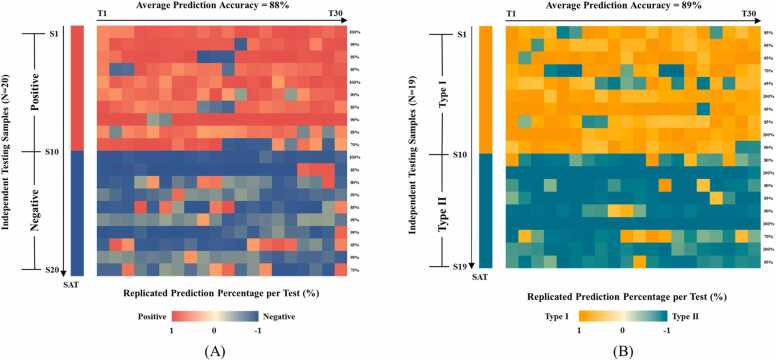
Validation of the CNN model performance. (A) Accuracy heatmap for identification of Hp-positive and Hp-negative serum samples. Red squares represent Hp-positive samples; blue squares represent Hp-negative samples. (B) Accuracy heatmap for identification of Type I and Type II Hp-positive serum samples. Orange squares represent Type I Hp-infection, while green squares define Type II Hp-infection. Color intensities represent the level of accuracy.

To validate the performance of our testing model and extend this method to new clinical settings for the identification of *H. pylori* infection and serum antibody types in blind samples, Fig. 6 presents the results of applying our best diagnostic model in this new clinical setting. Fig. 6**A** depicts the accuracy heatmap of the CNN model in identifying unknown infection status, with an average identification accuracy of 88 %. In Fig. 6**B**, the accuracy heatmap of the CNN model in identifying unknown antibody types was presented, with an average identification accuracy of 89 %. The color intensity of the squares in the figure represents the model's prediction probability for the SERS signals. In the blind test dataset, although some samples were misclassified, most samples showed high recognition accuracy, affirming the robustness and reliability of our approach. Additionally, qualitative examples of correctly and incorrectly classified spectra are provided in Supplementary Fig. S2.

### 3.7 Standalone analytical software

**Fig. 7:**
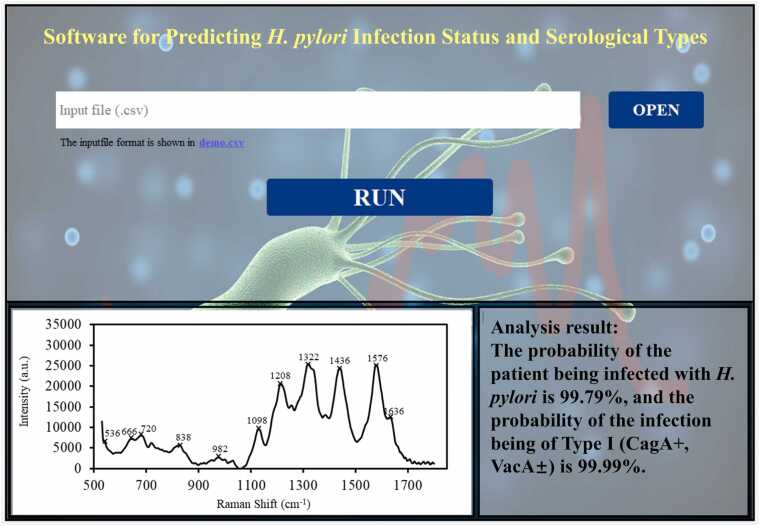
The GUI of the standalone software for rapid and accurate discrimination and prediction of *H. pylori* infection status and serological types based on the analysis of SERS spectra of human serum samples.

In this study, we developed standalone software that enables the prediction of SERS spectra from serum samples. The software includes a user-friendly graphical user interface (GUI), as depicted in Fig. 7. This interface lets users click the "OPEN" button to input their unknown SERS spectra. The software will then automatically visualize the spectra and generate characteristic peaks. The "RUN" button incorporates the CNN model to predict the infection status and typing of the input serum SERS. It should be noted that because there are potential system deviations between different Raman spectrometers, the current software can only detect the spectral data generated by the handheld Anton Paar™ Cora100 Raman spectrometer.

## 4 Discussion

*Helicobacter pylori* infection remains a significant global public health problem, particularly severe in developing countries. Several *H. pylori* virulence factors are important in its pathogenesis [4]. In particular, CagA and VacA are two toxin proteins that are closely associated with gastric carcinogenesis [46]. Conventional methods for detecting *H. pylori* infection status and antibody typing in serum, such as ELISA and Western blot, rely on specialized personnel and expensive equipment. In some resource-limited settings, these tests may not be feasible [47]. Therefore, there is an urgent need to develop a simple, economical, rapid, and accurate method for identifying *H. pylori* infection and antibody typing.

In this study, we developed a rapid and accurate detection method for determining *H. pylori* serostatus and identifying serum samples with immune responses to CagA and VacA using Raman spectroscopy and machine learning algorithms. SERS can provide detailed information about the chemical composition of tissues at the molecular level. Using noble metal nanoparticles (silver, gold, and copper), SERS is an effective tool for analyzing the vibrational properties of analytes at low concentrations down to the single molecule level [48]. In addition, it can identify unique fingerprint regions in individual cells and tissues, making it a promising clinical diagnostic tool [49], [50]. We obtained information about the *H. pylori* serostatus and antibody typing of all serum samples using quantum dot immunofluorescence. Based on the serological test results, an initial classification of the modeled samples was completed. The spectra were first preprocessed to transform the raw SERS spectral features into a feature matrix, which was then used as input to a machine-learning predictive model to detect *H. pylori* serostatus (Hp-positive, Hp-negative) and to discriminate Hp-positive serum samples (carcinogenic Type I, non-carcinogenic Type II). Our results demonstrated that simple average SERS spectral analysis did not discriminate biological samples due to significant variations in characteristic peaks [51]. Therefore, SERS spectroscopy for differentiating *H. pylori* serostatus and profiling immune responses of Hp-positive samples is challenging due to the complex composition of serum samples. To overcome this limitation, we used deconvolution spectra based on fine molecular vibrations, shown in previous studies, to help detect subtle differences between samples [52], [53]. As shown in Fig. 1, Fig. 2, these deconvolution peaks explained the subsequent decision-making process of supervised learning algorithms for serum sample classification.

Although much effort is put into removing undesirable effects during Raman spectroscopy measurements, such as normalization, curve smoothing, and baseline correction, the main challenge remains that the spectral signal is subject to many additional contributions from the instrument or the sample. So, we must do our best to mitigate the effects of live elimination when analyzing the data. Overlapping of SERS sample points occurs in the OPLS-DA clustering algorithm, distinguishing between Hp-negative and Hp-positive samples and differentiating Type I and II Hp-positive samples (Fig. 3). Therefore, there is a need to explore advanced Raman spectroscopy data analysis methods. Ho et al. pioneered integrating SERS with artificial intelligence algorithms to identify 30 common pathogenic bacteria [54]. Building on our previous research, in which we demonstrated the applicability of machine learning in diagnosing pathogenic microbes [33] and body fluid samples [55], we constructed six integrated learning methods using decision tree algorithms as weak classifiers. These models, including CNN, RF, SVM, AdaBoost, LDA, and DT, were trained using preprocessed spectral data as the input. Next, tuning the hyperparameters becomes an essential task for machine learning models. The primary task is to find the best combination of hyperparameters to obtain the best predictive model. Common types of hyperparameters include (i) κ in κ-NN, (ii) regularization constant, kernel type, and constants in SVMs, and (iii) number of layers, number of units per layer, and regularization in a neural network [56]. Tuning methods such as grid search, random search, and Bayesian optimization can be used to find the optimal values of the hyperparameters. In this study, we analyzed the quality of the hyperparametric combinations using a grid search approach. Each model’s parameter combination with the highest final score was selected to examine the SERS data for all samples. Among the six diagnostic models, the CNN model showed high predictive accuracy for diagnosing *H. pylori* serostatus (88.15 %) and for immune response typing (90.46 %). To further confirm the diagnostic performance of the different models, we used ROC curves to assess the specificity and sensitivity of each model, as well as the area under the curve (AUC) values to quantify the overall performance, and the results indicated that the CNN model was the most effective. Still, it is worth noting that this model also suffered from misclassification. In addition, we additionally collected 39 external validation samples to assess the performance of the CNN model under double-anonymized testing. The results showed that the model effectively identified unknown infection statuses (88 %) and immune response typing (89 %).

Although the CNN algorithm achieved satisfactory results in this study, there are still some things that could be improved. First, the infection status of the participants included in this study was confirmed by serological methods, and the current infection status of the patients remains to be confirmed. Second, although the study successfully distinguished different carcinogenic and non-carcinogenic Hp-positive infections based on SERS signals, the changes in serum involve alterations in various host proteins and signaling pathways. The specific reasons SERS can differentiate between different serum subtype samples still need further exploration. Additionally, the number of participants in the study cohort was relatively small, limiting the model’s ability to thoroughly learn the internal patterns of the same type of SERS signals. Larger-scale cohort studies could effectively enhance the model’s generalization capability. Furthermore, the model and independent software developed in this study were based on the Anton Paar™ Cora 100 Raman spectrometer, and their cross-platform capability and generalizability are limited. Developing analysis software for multi-platform SERS signals will be a focus of our future research. In conclusion, our work promotes the clinical application of the SERS-CNN method. The analysis software developed based on the Anton Paar™ Cora 100 provides a simple and convenient tool for analyzing SERS signals, providing a valuable and practical tool for healthcare professionals.

## 5 Conclusion

In conclusion, this study successfully demonstrated the potential of using the SERS spectra to diagnose *H. pylori* serostatus and differentiate serum samples with immune responses to CagA and VacA. Combined with CNN, we achieved robust classification results for serum SERS spectra. We also validated our prediction model efficacy by conducting double-anonymized experiments. Although our model showed a slight decline in sensitivity, we achieved significantly higher diagnostic accuracies of 80 % and 86 % for predicting serostatus of *H. pylori* infections and the immune response typing of Hp-positive serum samples, respectively. We also developed user-friendly software for spectral data generated by an Anton Paar™ Cora100 handheld Raman spectrometer that can predict *H. pylori* infection status and toxins-related immune response typing. Like other serological tests, our diagnostic technique also has the limitation of distinguishing between a current and past *H. pylori* infection. However, when combined with other diagnostic tests, such as UBT and qPCR, it can be a perfect diagnostic tool for screening carcinogenic *H. pylori* infection at the population level. This novel deep learning-based serum SERS intelligent analysis model holds a significant potential to positively impact clinical practice, particularly in settings with limited resources where it is crucial to have cost-effective and efficient methods for screening *H. pylori* infection and assessing its associated risks. Our study significantly contributes to the ongoing efforts to address health challenges related to *H. pylori*. By offering a robust and easily accessible diagnostic tool, we move closer to enhancing the management and prevention of the detrimental outcomes caused by this pathogen, specifically its correlation with gastric cancer.

## Ethical approval

Informed consent was obtained from all participants involved in this study. Acquisition of serum samples was carried out following the approval of the Ethics Committee at the Fifth People’s Hospital of Huai’an (No. HAWY-KY-2023–020-01). All experiments strictly adhered to the guidelines and regulations set forth by the Ethics Committee at the Fifth People’s Hospital of Huai’an, Jiangsu Province, China.

## Funding statement

This study was supported by the Guangdong Basic and Applied Basic Research Foundation [Grant Nos. 2022A1515220023, 2022B1515230005], Research Foundation for Advanced Talents of Guandong Provincial People’s Hospital [Grant Nos. KY012023293, KJ012021097], 10.13039/501100001809National Natural Science Foundation of China [Grant Nos. 82272423, 82072380], 10.13039/501100012166National Key Research and Development Program of China [Grant No. 2023YFC2606200], Guangzhou Key Research and Development Program [Grant No. 2023B03J1248], and The Fifth People’s Hospital of Huai'an Collaboration Foundation [Grant Nos. HWY-YL-20230072, HAWY-KY-YNKT-202301].

## Author statement

We did not use any generative AI or AI-assisted technologies in the preparation of this work.

## CRediT authorship contribution statement

**Xue-Song Xiong:** Writing – original draft, Visualization, Methodology, Data curation. **Jin-Ting Wang:** Writing – review & editing, Software, Methodology, Data curation. **Liang Wang:** Writing – review & editing, Writing – original draft, Supervision, Resources, Investigation, Funding acquisition, Conceptualization. **Jia-Wei Tang:** Writing – review & editing, Writing – original draft, Visualization, Validation, Software, Methodology, Investigation, Formal analysis, Data curation. **Zeeshan Umar:** Writing – review & editing, Writing – original draft, Visualization, Software, Data curation. **Fen Li:** Writing – review & editing, Methodology, Investigation, Conceptualization. **Wei-Xuan Yang:** Writing – review & editing, Supervision, Investigation, Conceptualization. **Yu-Ting Si:** Writing – review & editing, Writing – original draft, Visualization, Validation, Formal analysis, Data curation. **Bing Gu:** Writing – review & editing, Supervision, Methodology, Investigation, Conceptualization. **Li Zhang:** Writing – review & editing, Supervision, Methodology, Conceptualization. **Barry J. Marshall:** Writing – review & editing, Supervision, Methodology, Investigation, Conceptualization. **Alfred Chin Yen Tay:** Writing – review & editing, Supervision, Resources, Investigation, Conceptualization. **Eng Guan Chua:** Writing – review & editing, Investigation. **Quan Yuan:** Writing – original draft, Visualization, Data curation.

## Declaration of Competing Interest

The authors declare that the research was conducted in the absence of any commercial or financial relationships that could be construed as a potential conflict of interest.
