# Supplementary material for: Rapid profiling of carcinogenic types of Helicobacter pylori infection via deep learning analysis of label-free SERS spectra of human serum
Source: Comput Struct Biotechnol J. 2024 Sep 16;23:3379–90. doi: 10.1016/j.csbj.2024.09.008 (PMC11424770; doi:10.1016/j.csbj.2024.09.008)
Supplement: Supplementary file 1 — Supplementary material [file mmc1.docx]

**Supplementary Table S1** Baseline information on participants in model training for Hp-positive and Hp-negative serum samples

|  | **Positive**  **(n = 50)** | **Negative**  **(n = 50)** |
| --- | --- | --- |
| **Median age, year (range)** | **47 (26-60)** | **41 (20-58)** |
| **18 ≤ Age ≤ 29, n (%)** | **1 (2)** | **10 (20)** |
| **30 ≤ Age ≤ 39, n (%)** | **11 (22)** | **14 (28)** |
| **40 ≤ Age ≤ 49, n (%)** | **12 (24)** | **8 (16)** |
| **50 ≤ Age ≤ 60, n (%)** | **26 (52)** | **18 (36)** |
| **Gender, n (%)** |  |  |
| **Male** | **19 (38)** | **17 (34)** |
| **Female** | **31 (62)** | **33 (66)** |
| **Median BMI, (range)** |  |  |
| **Underweight (BMI < 18.5), n (%)** | **0 (0)** | **0 (0)** |
| **Normal weight (18.5 ≤ BMI ≤ 24.9), n (%)** | **27 (54)** | **29 (58)** |
| **Overweight (25.0 ≤ BMI ≤ 29.9), n (%)** | **23 (46)** | **18 (36)** |
| **Obesity (BMI ≥ 30), n (%)** | **0 (0)** | **3 (6)** |

**Supplementary Table S2** Baseline information on participants in model training for Type I and Type II Serum Samples

|  | **Type I**  **(n = 25)** | **Type II**  **(n = 25)** |
| --- | --- | --- |
| **Median age, year (range)** | **47 (26-60)** | **48 (32-58)** |
| **18 ≤ Age ≤ 29, n (%)** | **1 (4)** | **0 (0)** |
| **30 ≤ Age ≤ 39, n (%)** | **5 (20)** | **6 (24)** |
| **40 ≤ Age ≤ 49, n (%)** | **7 (28)** | **5 (20)** |
| **50 ≤ Age ≤ 60, n (%)** | **12 (48)** | **14 (56)** |
| **Gender, n (%)** |  |  |
| **Male** | **11 (44)** | **8 (32)** |
| **Female** | **14 (56)** | **17 (68)** |
| **Median BMI, (range)** |  |  |
| **Underweight (BMI < 18.5), n (%)** | **0 (0)** | **0 (0)** |
| **Normal weight (18.5 ≤ BMI ≤ 24.9), n (%)** | **15 (60)** | **12 (48)** |
| **Overweight (25.0 ≤ BMI ≤ 29.9), n (%)** | **10 (40)** | **13 (52)** |
| **Obesity (BMI ≥ 30), n (%)** | **0 (0)** | **0 (0)** |

**Supplementary Table S3** Best Combination of Hyperparameter for Different Machine Learning Algorithms on Hp-positive and Hp-negative Serum Samples Classification Task.

| **Algorithms** | **Parameter Range** | **Optimum Parameter** |
| --- | --- | --- |
| **AdaBoost** | **learning_rate** = [0.1, 1, 0.01, 0.001],  **n_estimators** = [50, 60, 70, 80, 90, 100, 110, 120, 130, 140, 150, 160, 170, 180] | **learning_rate** = 1, **n_estimators** = 185 |
| **Decision Tree** | **criterion** = ['gini', 'entropy'],  **max_depth** = range (1, 30), **max_features** = [21, 22, 23, 24, 25, 26, 28, 29, 30, 'auto'] | **criterion** = ' entropy',  **max_depth** = 27,  **max_features** = 38 |
| **Linear Discrimination**  **Analysis** | **solver =** ['eigen', 'lsqr']  **shrinkage =** [0.01, 0.1, 0.2, 0.3, 0.4, 0.5, 0.6, 0.7, 0.8, 0.9, 'auto'] | **Solver =** ' eigen',  **shrinkage =** 'auto' |
| **Random Forest** | **Criterion** = ['gini', 'entropy'],  **max_depth** = range (1, 10),  **n_estimators** = [50, 60, 70, 80, 90, 100, 110, 120, 130, 140, 150, 160, 170, 180] | **criterion** = 'gini', **max_depth** = 29, **n_estimators** = 150 |
| **SVM** | **Cs =** [0.0001, 0.001, 0.01, 0.1, 1, 2, 3, 4, 5, 10],  **gamma =** [0.0001, 0.001, 0.01, 0.1, 1],  kernel = ['rbf', ' linear '] | **Cs =** 0.0001,  **gamma =** 0.0001,  **kernel =** 'linear',  **probability =** True |

**Supplementary Table S4** Best Combination of Hyperparameters for Different Machine Learning Algorithms on Type I and II Serum Samples Classification Task.

| **Algorithms** | **Parameter Range** | **Optimum Parameter** |
| --- | --- | --- |
| **AdaBoost** | **learning_rate** = [0.1, 1, 0.01, 0.001],  **n_estimators** = [50, 60, 70, 80, 90, 100, 110, 120, 130, 140, 150, 160, 170, 180] | **learning_rate** = 1, **n_estimators** = 175 |
| **Decision Tree** | **criterion** = ['gini', 'entropy'],  **max_depth** = range (1, 30), **max_features** = [21, 22, 23, 24, 25, 26, 28, 29, 30, 'auto'] | **criterion** = ' entropy',  **max_depth** = 6,  **max_features** = 36 |
| **Linear Discrimination**  **Analysis** | **solver =** ['eigen', 'lsqr']  **shrinkage =** [0.01, 0.1, 0.2, 0.3, 0.4, 0.5, 0.6, 0.7, 0.8, 0.9, 'auto'] | **Solver =** ' eigen',  **shrinkage =** 'auto' |
| **Random Forest** | **Criterion** = ['gini', 'entropy'],  **max_depth** = range (1, 10),  **n_estimators** = [50, 60, 70, 80, 90, 100, 110, 120, 130, 140, 150, 160, 170, 180] | **criterion** = 'gini', **max_depth** = 25, **n_estimators** = 100 |
| **SVM** | **Cs =** [0.0001, 0.001, 0.01, 0.1, 1, 2, 3, 4, 5, 10],  **gamma =** [0.0001, 0.001, 0.01, 0.1, 1],  kernel = ['rbf', ' linear '] | **Cs =** 0.0001,  **gamma =** 0.0001,  **kernel =** 'linear',  **probability =** True |

**Supplementary Table S5** Band assignments of characteristic peaks to potential metabolites in SERS spectra of Hp-positive and Hp-negative serum samples.

| **Wavenumber (cm-1)** | **Band Assignment** | **Negative** | **Positive** | ***Ref.*** |
| --- | --- | --- | --- | --- |
| 676 | Myeloperoxidase |  |  | 1 |
| 826 | Nucleic acids |  |  | 2 |
| 1128 | C-O stretching |  |  | 3 |
| 1216 | Amide III |  |  | 4 |
| 1258 | C-N stretching vibration |  |  | 5 |
| 1316 | Guanine |  |  | 6 |
| 1320 | Guanine |  |  | 7 |
| 1338 | CH_3_CH_2_ wagging |  |  | 8 |
| 1434 | CH_2_ deformation in protein and lipid |  |  | 9 |
| 1588 | C double bond C single bond C stretching in Adenine |  |  | 10 |

**Reference**

[1] K. Naseer, M. Saleem, S. Ali, B. Mirza, J.J.S.A.P.A.M. Qazi, B. Spectroscopy, Identification of new spectral signatures from hepatitis C virus-infected human sera, 222 (2019) 117181.

[2] H. Liang, X. Kong, Y. Ren, H. Wang, E. Liu, F. Sun, G. Zhu, Q. Zhang, Y.J.S.A.P.A.M. Zhou, B. Spectroscopy, Application of Serum Raman Spectroscopy in Rapid and Early Discrimination of Aplastic Anemia and Myelodysplastic Syndrome, (2023) 123008.

[3] J.A.M. Bispo, E.E. de Sousa Vieira, L. Silveira Jr, A.B.J.J.o.b.o. Fernandes, Correlating the amount of urea, creatinine, and glucose in urine from patients with diabetes mellitus and hypertension with the risk of developing renal lesions using Raman spectroscopy and principal component analysis, 18(8) (2013) 087004-087004.

[4] M.A. Ochsenkuhn, P.R. Jess, H. Stoquert, K. Dholakia, C.J.J.A.N. Campbell, Nanoshells for surface-enhanced Raman spectroscopy in eukaryotic cells: cellular response and sensor development, 3(11) (2009) 3613-3621.

[5] K. Ock, W.I. Jeon, E.O. Ganbold, M. Kim, J. Park, J.H. Seo, K. Cho, S.-W. Joo, S.Y.J.A.c. Lee, Real-time monitoring of glutathione-triggered thiopurine anticancer drug release in live cells investigated by surface-enhanced Raman scattering, 84(5) (2012) 2172-2178.

[6] X. Zhang, X. Song, W. Li, C. Chen, M. Wusiman, L. Zhang, J. Zhang, J. Lu, C. Lu, X.J.S.R. Lv, Rapid diagnosis of membranous nephropathy based on serum and urine Raman spectroscopy combined with deep learning methods, 13(1) (2023) 3418.

[7] A. Dutta, R. Gautam, S. Chatterjee, F. Ariese, S.K. Sikdar, S.J.A.c.n. Umapathy, Ascorbate protects neurons against oxidative stress: a Raman microspectroscopic study, 6(11) (2015) 1794-1801.

[8] Y. Lin, S. Gao, M. Zheng, S. Tang, K. Lin, S. Xie, Y. Yu, J.J.S.A.P.A.M. Lin, B. Spectroscopy, A microsphere nanoparticle based-serum albumin targeted adsorption coupled with surface-enhanced Raman scattering for breast cancer detection, 261 (2021) 120039.

[9] X. Cao, C. Shi, W. Lu, H. Zhao, M. Wang, W. Tong, J. Dong, X. Han, W.J.J.o.N. Qian, Nanotechnology, Synthesis of Au nanostars and their application as surface-enhanced Raman scattering-activity tags inside living cells, 15(7) (2015) 4829-4836.

[10] M. Kashif, M.I. Majeed, M.A. Hanif, A.J.S.A.P.A.M. ur Rehman, B. Spectroscopy, Surface Enhanced Raman Spectroscopy of the serum samples for the diagnosis of Hepatitis C and prediction of the viral loads, 242 (2020) 118729.

**Supplementary Table S6** Band assignments of characteristic peaks to potential metabolites in SERS spectra of Type I and II serum samples.

| **Wavenumber (cm-1)** | **Band Assignment** | **Type I** | **Type II** | ***Ref.*** |
| --- | --- | --- | --- | --- |
| 676 | Myeloperoxidase |  |  | 1 |
| 826 | Nucleic acids |  |  | 2 |
| 1128 | C-O stretching |  |  | 3 |
| 1216 | Amide III |  |  | 4 |
| 1258 | C-N stretching vibration |  |  | 5 |
| 1280 | Amide III |  |  | 6 |
| 1318 | Guanine |  |  | 7 |
| 1322 | Guanine |  |  | 8 |
| 1338 | CH_3_CH_2_ wagging |  |  | 9 |
| 1434 | CH_2_ deformation in protein and lipid |  |  | 10 |
| 1588 | C double bond C single bond C stretching in Adenine |  |  | 11 |

**Reference**

[1] K. Naseer, M. Saleem, S. Ali, B. Mirza, J.J.S.A.P.A.M. Qazi, B. Spectroscopy, Identification of new spectral signatures from hepatitis C virus infected human sera, 222 (2019) 117181.

[2] H. Liang, X. Kong, Y. Ren, H. Wang, E. Liu, F. Sun, G. Zhu, Q. Zhang, Y.J.S.A.P.A.M. Zhou, B. Spectroscopy, Application of Serum Raman Spectroscopy in Rapid and Early Discrimination of Aplastic Anemia and Myelodysplastic Syndrome, (2023) 123008.

[3] J.A.M. Bispo, E.E. de Sousa Vieira, L. Silveira Jr, A.B.J.J.o.b.o. Fernandes, Correlating the amount of urea, creatinine, and glucose in urine from patients with diabetes mellitus and hypertension with the risk of developing renal lesions using Raman spectroscopy and principal component analysis, 18(8) (2013) 087004-087004.

[4] M.A. Ochsenkuhn, P.R. Jess, H. Stoquert, K. Dholakia, C.J.J.A.N. Campbell, Nanoshells for surface-enhanced Raman spectroscopy in eukaryotic cells: cellular response and sensor development, 3(11) (2009) 3613-3621.

[5] K. Ock, W.I. Jeon, E.O. Ganbold, M. Kim, J. Park, J.H. Seo, K. Cho, S.-W. Joo, S.Y.J.A.c. Lee, Real-time monitoring of glutathione-triggered thiopurine anticancer drug release in live cells investigated by surface-enhanced Raman scattering, 84(5) (2012) 2172-2178.

[6] A. Sahu, N. Nandakumar, S. Sawant, C.M.J.A. Krishna, Recurrence prediction in oral cancers: a serum Raman spectroscopy study, 140(7) (2015) 2294-2301.

[7] R.V. John, T. Devasia, M. N, J. Lukose, S.J.L.i.M.S. Chidangil, Micro-Raman spectroscopy study of blood samples from myocardial infarction patients, 37(9) (2022) 3451-3460.

[8] Y. Xu, C.J.S.i.C.S.C.L.S. Lu, Raman spectroscopic study on the structure of human immunodeficiency virus (HIV) and hypericin-induced photosensitive damage of HIV, 48 (2005) 117-132.

[9] Y. Lin, S. Gao, M. Zheng, S. Tang, K. Lin, S. Xie, Y. Yu, J.J.S.A.P.A.M. Lin, B. Spectroscopy, A microsphere nanoparticle based-serum albumin targeted adsorption coupled with surface-enhanced Raman scattering for breast cancer detection, 261 (2021) 120039.

[10] X. Cao, C. Shi, W. Lu, H. Zhao, M. Wang, W. Tong, J. Dong, X. Han, W.J.J.o.N. Qian, Nanotechnology, Synthesis of Au nanostars and their application as surface enhanced Raman scattering-activity tags inside living cells, 15(7) (2015) 4829-4836.

[11] M. Kashif, M.I. Majeed, M.A. Hanif, A.J.S.A.P.A.M. ur Rehman, B. Spectroscopy, Surface Enhanced Raman Spectroscopy of the serum samples for the diagnosis of Hepatitis C and prediction of the viral loads, 242 (2020) 118729.

**Supplementary Table S7** Baseline information on participants in model validation for Hp-positive and Hp-negative serum samples

|  | **Positive**  **(n = 10)** | **Negative**  **(n = 10)** |
| --- | --- | --- |
| **Median age, year (range)** | **48 (24-60)** | **40 (23-58)** |
| **18 ≤ Age ≤ 29, n (%)** | **1 (10)** | **2 (20)** |
| **30 ≤ Age ≤ 39, n (%)** | **1 (10)** | **3 (30)** |
| **40 ≤ Age ≤ 49, n (%)** | **3 (30)** | **2 (20)** |
| **50 ≤ Age ≤ 60, n (%)** | **5 (50)** | **3 (30)** |
| **Gender, n (%)** |  |  |
| **Male** | **6 (60)** | **5 (50)** |
| **Female** | **4 (40)** | **5 (50)** |
| **Median BMI, (range)** |  |  |
| **Underweight (BMI < 18.5), n (%)** | **0 (0)** | **0 (0)** |
| **Normal weight (18.5 ≤ BMI ≤ 24.9), n (%)** | **9 (90)** | **4 (40)** |
| **Overweight (25.0 ≤ BMI ≤ 29.9), n (%)** | **1 (10)** | **5 (50)** |
| **Obesity (BMI ≥ 30), n (%)** | **0 (0)** | **1 (10)** |

**Supplementary Table S8** Baseline information on participants in model validation for Type I and Type II Serum Samples

|  | **Type I**  **(n = 10)** | **Type II**  **(n = 9)** |
| --- | --- | --- |
| **Median age, year (range)** | **44 (24-60)** | **50 (39-58)** |
| **18 ≤ Age ≤ 29, n (%)** | **1 (10)** | **0 (0)** |
| **30 ≤ Age ≤ 39, n (%)** | **2 (20)** | **1 (11)** |
| **40 ≤ Age ≤ 49, n (%)** | **4 (40)** | **3 (33)** |
| **50 ≤ Age ≤ 60, n (%)** | **3 (30)** | **5 (56)** |
| **Gender, n (%)** |  |  |
| **Male** | **2 (20)** | **4 (44)** |
| **Female** | **8 (80)** | **5 (56)** |
| **Median BMI, (range)** |  |  |
| **Underweight (BMI < 18.5), n (%)** | **0 (0)** | **0 (0)** |
| **Normal weight (18.5 ≤ BMI ≤ 24.9), n (%)** | **6 (60)** | **6 (67)** |
| **Overweight (25.0 ≤ BMI ≤ 29.9), n (%)** | **4 (40)** | **3 (33)** |
| **Obesity (BMI ≥ 30), n (%)** | **0 (0)** | **0 (0)** |


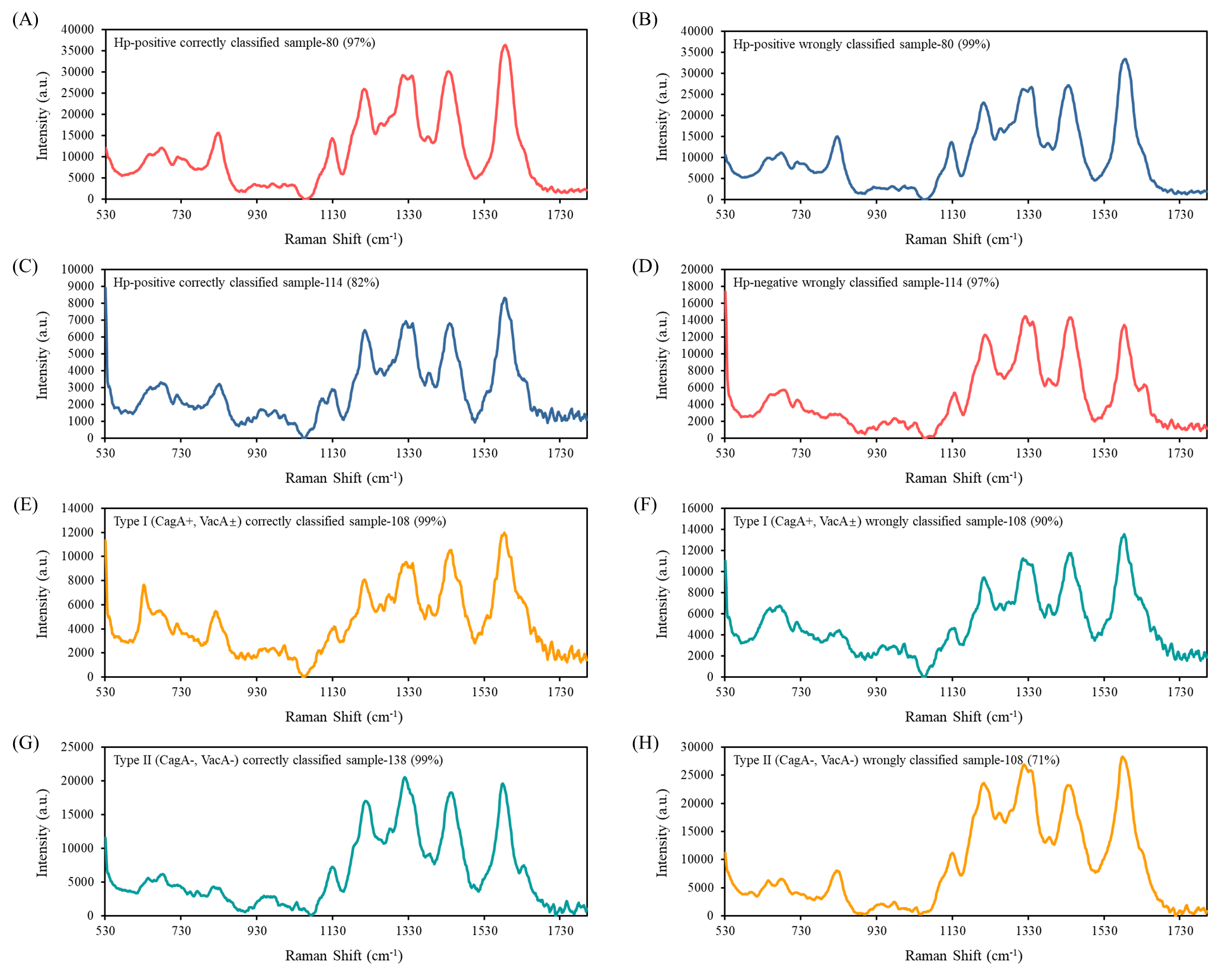


**Supplementary Figure S1** Qualitative examples of correctly and wrongly classified spectra. The percentage represents the model's prediction accuracy for the spectrum. The X-axis represents Raman shifts in the 530-1800 cm^-1^ range, while the Y-axis represents the relative Raman intensity. a.u. is an arbitrary unit, referring to the relative value of each data under the same measurement conditions.
