# Supplementary material for: Rapid profiling of carcinogenic types of Helicobacter pylori infection via deep learning analysis of label-free SERS spectra of human serum
Source: Comput Struct Biotechnol J. 2024 Sep 16;23:3379–90. doi: 10.1016/j.csbj.2024.09.008 (PMC11424770; doi:10.1016/j.csbj.2024.09.008)
Supplement: Supplementary file 2 — Supplementary material [file mmc2.docx]

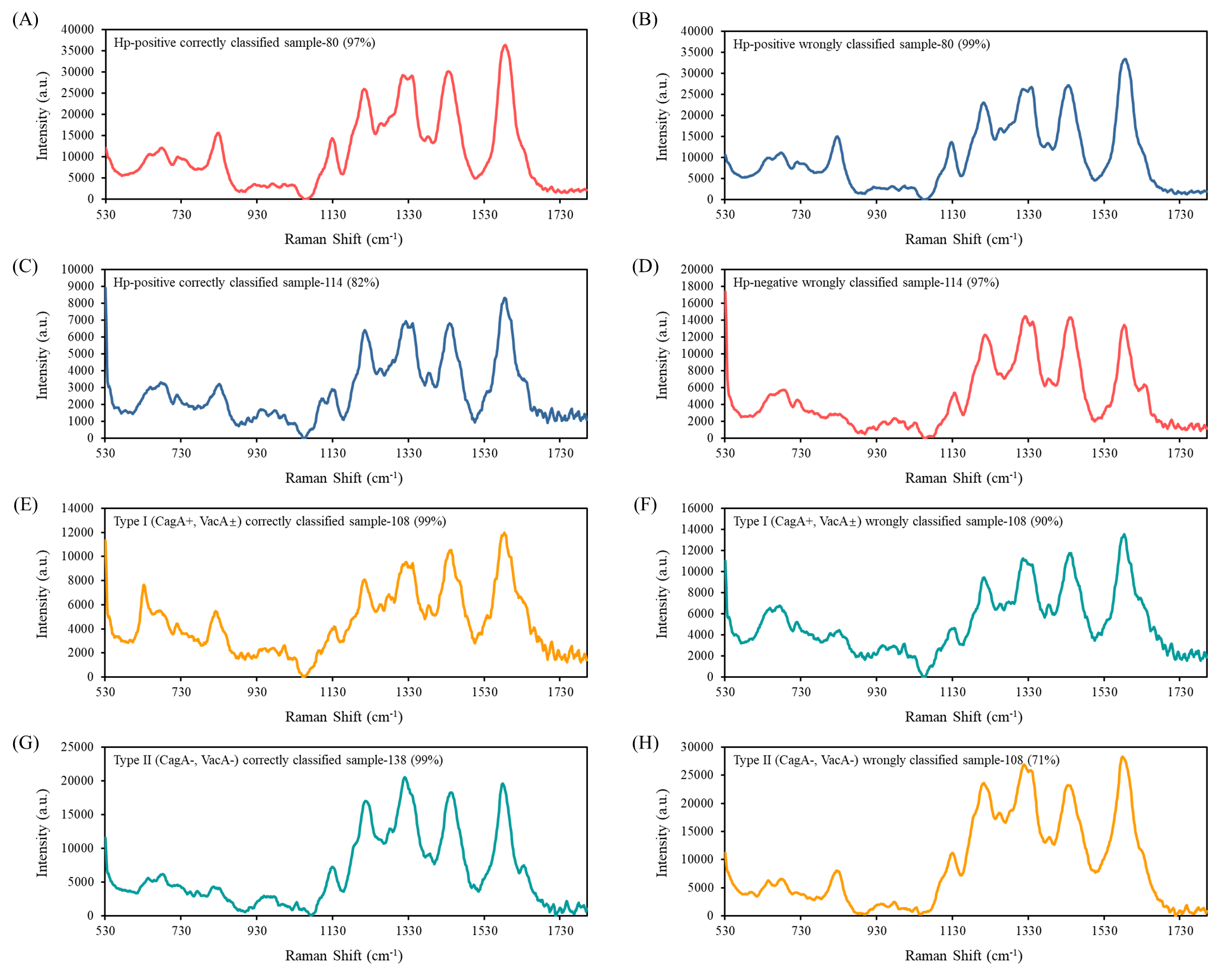


**Supplementary Figure S1** Qualitative examples of correctly and wrongly classified spectra. The percentage represents the model's prediction accuracy for the spectrum. X-axis represents Raman shifts in the 530-1800 cm^-1^ range, while the Y-axis represents the relative Raman intensity. a.u. is an arbitrary unit, referring to the relative value of each data under the same measurement conditions.
