# Supplementary material for: Rapid profiling of carcinogenic types of Helicobacter pylori infection via deep learning analysis of label-free SERS spectra of human serum
Source: Comput Struct Biotechnol J. 2024 Sep 16;23:3379–90. doi: 10.1016/j.csbj.2024.09.008 (PMC11424770; doi:10.1016/j.csbj.2024.09.008)
Supplement: Supplementary file 3 — Supplementary material [file mmc3.docx]

**Average spectrum baseline correction**

Each type of average spectrum was saved as a .txt file, with each entry consisting of two columns, including Raman shift and intensity. The data were inputed into the Raman spectroscopy analysis software LabSpec (Version 5.58.25) for baseline correction. The "Scale normalization" option was selected to align the data, followed by the "baseline" function with an "Auto" fit applied to the data. The resulting data were saved as .txt files for subsequent deconvolution analysis using Origin software.

**Spectral deconvolution and characteristic peaks**

For the average SERS signals of *H. pylori* infection negative and positive patients, as well as type I and type II patients, the analysis was performed using Origin software (version 2019b, OriginLab, United States). Specifically, the baseline-corrected spectral data were input, and the analysis was started by selecting "Analysis -> Peaks and Baseline -> Peak Analyzer". After fitting peaks using "Fit Peaks (Pro)", the "Find" operation was executed to identify spectral feature peaks. Spectral feature peaks were controlled using "Add" and "Modify/Del", and deconvolution spectral signal fitting was performed using "Fit Control". The "Vogit" linear function was used to extract spectral information of related peaks, which is the convolution of Lorentzian and Gaussian density functions. In this function, the half-widths of the Lorentzian and Gaussian linear functions were considered, and the comprehensive widened linear function V (v, v0) was obtained. Finally, spectral matching points were automatically filtered to find those closest to the experimental spectrum.

**Normalization**

The SERS signals with outliers removed were processed using the commercial analysis software Unscrambler X (Version 10.4 64bit, CAMO). Specifically, the "Transform" function was selected from the "Task" menu to perform "Normalize". In the "Matrix", each row represents a SERS signal, and each column represents a Raman shift. The "Type" was set to "Unit vector normalization", which scales the signal intensity to the range [0, 1] and eliminates overall intensity differences among spectra, ensuring that each spectrum has the same weight in the analysis.
